# Advancing cardiac motion estimation with emerging AI techniques for enhanced echocardiographic image registration

**DOI:** 10.1016/j.mex.2025.103432

**Published:** 2025-06-17

**Authors:** M. Rajesh, S. Balakrishnan, R. Elankavi

**Affiliations:** Department of Computer Science and Engineering, Aarupadai Veedu Institute of Technology, Vinayaka Mission’s Research Foundation (DU), Chennai, Tamil Nadu 603104, India

**Keywords:** Cardiac motion estimation, Image registration, Neural Radiance Fields, Diffusion Models, Self-supervised learning, HC-IoT Architecture (Holographic Counterpart - Internet of Things Architecture)

## Abstract

Monitoring and diagnosis of cardiovascular diseases rely on cardiac motion estimation. The methods used for registering echocardiographic images have drawbacks such as low resolution, noise, and distortion of the anatomy. In order to enhance the prediction of cardiac motion, this research presents an AI-powered architecture that makes use of Vision Transformers, Diffusion Models, and Neural Radiance Fields (NeRF). Adversarial and self-supervised contrastive learning enhance picture quality and generalisability across adult and foetal echocardiography, while a graph neural network (GNN)-based anatomical constraint maintains heart shape. Better, more accurate, more efficient real-time motion tracking without relying on massive labelled datasets is possible with the proposed approach. Cardiac motion analysis in a wide range of patient populations is now therapeutically viable, thanks to this innovative approach that improves echocardiographic picture registration.

• Utilizes Vision Transformers, Diffusion Models, and NeRF for high-quality cardiac motion prediction.
• Adversarial and self-supervised contrastive learning improve echocardiographic registration across demographics.
• A GNN-based anatomical constraint ensures accurate heart morphology during motion analysis.

## Graphical abstract

**Figure:**
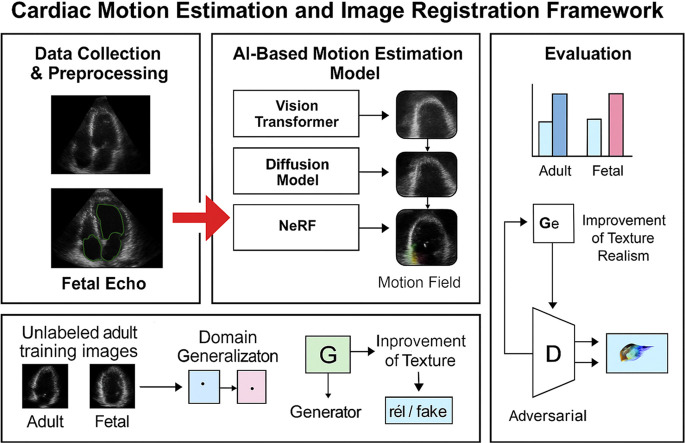

Specifications table

**Table:** 

Subject area:	Computer Science
More specific subject area:	AI-enabled Contactless Biosensing and Privacy-Preserving Health Monitoring for Ovulation Prediction
Name of your method:	HC-IoT Architecture (Holographic Counterpart - Internet of Things Architecture)
Name and reference of original method:	1. McMahan, B., et al. (2017). Communication-Efficient Learning of Deep Networks from Decentralized Data. AISTATS. https://arxiv.org/abs/1602.05629 2. Adib, F., et al. (2015). Smart homes that monitor breathing and heart rate. CHI Conference. https://doi.org/10.1145/2702123.2702200 3. Miotto, R., et al. (2016). Deep Patient: An Unsupervised Representation to Predict the Future of Patients from the Electronic Health Records. Scientific Reports. https://doi.org/10.1038/srep26094
Resource availability:	Hardware: Radar sensors, PPG modules, LiDAR systems, AI-capable edge devices Software: Federated learning frameworks, multimodal AI toolkits, signal processing tools Manpower: Experts in AI, biosignal processing, federated learning, embedded systems Scalability: Validated in edge-based setups; adaptable to mobile/wearable devices

## Background

Cardiovascular illnesses are the major cause of death worldwide, requiring accurate and early non-invasive imaging diagnosis. Echocardiography is popular because to its portability, cost-effectiveness, and real-time imaging. Echocardiographic pictures have low spatial resolution, speckle noise, and anatomical distortions, especially in dynamic sequences, making heart motion assessment difficult. Handcrafted features and supervised learning methods in traditional picture registration and motion tracking require large annotated datasets, making them unsuitable for clinical use.

Medical image analysis has changed due to deep learning and other AI breakthroughs. Vision Transformers, Diffusion Models, and Neural Radiance Fields (NeRF) enable sophisticated spatial and temporal pattern comprehension with minimum supervision. Self-supervised and adversarial learning offer robust generalisation across adult and foetal echocardiographic datasets. A structural precondition for morphological fidelity during motion estimation is graph neural networks (GNNs) with anatomical constraints. These new AI methods enable high-precision, real-time cardiac motion estimation and image registration with better clinical application.

## Method details

### Introduction

A precise assessment of cardiac motion is essential when diagnosing and monitoring cardiovascular disorders such as cardiomyopathy, heart failure, or congenital defects. Echocardiography generates real-time pictures, is non-invasive, and costs-effective. Motion estimation has the potential to identify cardiac issues; however, it could be hindered by imaging artefacts, speckle noise, and subpar image quality. It is common for optical flow and free-form deformation image registration technologies to harm the structure of the heart because of the high processing power requirements associated with them. One recent development is the use of deep learning for image registration (DLIR) specifically for evaluating cardiac motion [1]. It outperforms previous approaches in terms of efficiency and precision. The inability to adapt to foetal echocardiography, poor anatomical preservation, increasing data reliance, improved texture quality, and other issues limit the practical utility of DLIR approaches. When calculating cardiac motion using DLIR, it is challenging to preserve anatomical details [2]. Many models produce inaccurate clinical results because they overestimate cardiac characteristics due to a lack of global form limitations. Visual distortions occur when the models prioritise pixel-wise likeness above texture. Due to the large size of the foetal heart and interference from maternal tissues, most DLIR foetal imaging fails [3]. Adult echocardiography datasets are utilised to instruct these approaches. Tagged datasets aren't practical for use in real-time because of their size and expense. In light of these concerns, a registration system powered by AI is essential. Without sacrificing picture quality or anatomical detail, this new approach needs to be applicable to a wide range of patient types. Further, it needs to be functional [4].

Machine learning-powered picture registration is presented in this article. It employs diffusion models, vision transformers, and NeRF to circumvent these limitations. Graph neural networks (GNNs) enhance 3D motion tracking and picture alignment without modifying the structure of the heart by imposing anatomical constraints. Adversarial learning and self-supervised contrastive learning enhance both adult and foetal echocardiograms [5]. Self-supervised learning eliminates the necessity for extensive labelled datasets, facilitating rapid training on unlabelled echocardiographic recordings. This study is distinguished by its application of AI technologies for echocardiographic image registration [6]. Initially, NeRF and diffusion models were employed to generate high-fidelity pictures and characterise intricate deformations to assess cardiac velocity [7]. The GNN-based form restriction preserves structural integrity, while the adversarial texture refinement technique enhances visual perception. Regrettably, current DLIR algorithms cannot generate reliable motion estimates without human annotations. The issue has been resolved by the self-supervised contrastive learning architecture [8].

The detection, diagnosis, and treatment of cardiovascular illnesses such myocardial infarction, cardiomyopathy, and congenital heart defects are all aided by accurate cardiac motion analysis [9]. Clinicians can use it to assess mechanical synchrony, disease progression, therapeutic efficacy, and myocardial function. Since functional evaluation is not always possible in foetal and neonatal cardiology, accurate motion estimation might be helpful in making diagnoses. Although motion analysis has great clinical utility, its usage is limited due to imaging and processing limits. To address this issue, this research presents a strong architecture for echocardiographic motion tracking that is driven by artificial intelligence [10].

Echocardiographic imaging necessitates the challenging but essential task of estimating heart motion. While deep learning-based registration has enhanced motion tracking, the current models have limitations due to their reliance on huge labelled datasets and their distortion of heart architecture. In order to improve accuracy, efficiency, and generalisability, this study suggests an AI-driven framework that combines NeRF, Diffusion Models, GNN, and self-supervised learning. With the proposed method's much enhanced registration quality, adult and foetal echocardiography can now use real-time heart motion analysis for therapeutic purposes.

### Related Works

In order to identify cardiovascular illnesses and get insight into heart function, echocardiographic imaging uses cardiac motion estimation. It is challenging to track the heart's movement because to motion distortions, speckle noise, and poor picture quality. Optic flow and free-form deformation are two examples of traditional image registration methods that require a lot of processing power without preserving anatomy [1,2]. Researchers are looking at new artificial intelligence approaches, including deep learning (DL), to improve heart motion predictions because of these limitations. It is possible to damage cardiac components using traditional methods of estimating cardiac motion that rely on pixel-wise similarity [3]. Optical flow motion tracking pictures that are too blurry or have too little resolution can lead to incorrect myocardial deformations [4]. Although free-form deformation (FFD) registration is more versatile, it is more challenging to maintain anatomical integrity due to the utilisation of costly processing resources and pre-existing models [5].

There has been a notable uptick in the accuracy of heart motion estimate algorithms using deep learning. By faithfully depicting complicated anomalies in adult and foetal echocardiographic pictures, these methods enhance motion tracking [6,7]. Convolutional neural networks (CNNs) are frequently used for segmentation, motion estimation, and registration in pictures [8,9]. Deep learning algorithms can learn sophisticated relationships between cardiac phases in order to improve the accuracy of motion estimates on huge datasets. Cine magnetic resonance imaging (MRI) motion tracking has been improved using convolutional neural networks (CNNs) in terms of 3D motion estimation in the myocardium and endocardium [10,11]. It is essential for clinical diagnosis that the anatomical plausibility of registered pictures be preserved, but many deep learning systems have difficulty achieving this, even when they have the potential to do so [12].

Maintaining anatomical structures is one of the most significant difficulties associated with picture registration that uses deep learning. Pixel-wise similarity is prioritised over the structural integrity of cardiac tissues, such as the myocardium, in several models [13]. Recently, deep learning models have been given anatomical limitations in order to maintain the heart's form. Graph neural networks (GNNs) can be used to represent the spatial relationships between cardiac areas, which helps ensure that registered images are structurally consistent [14,15]. This maintains the structure of the heart while enhancing the accuracy of motion estimation. Adversarial learning has improved the quality of pictures, which has in turn improved cardiac structural texturing [16]. Adversarial networks, and GANs in particular, produce more realistic and higher-fidelity outcomes in cardiac image registration [17]. Although deep learning models have been successful with adult echocardiograms, specialised procedures are required for echocardiograms of foetuses. Foetal echocardiogram is difficult because the foetal heart is so little that the pictures are of low quality, and the motion estimate is affected by maternal tissue [18]. Recent study has shown that models trained on datasets of foetal echocardiography are needed to overcome these hurdles. The absence of large annotated datasets is one of the challenges that must be overcome in order to create deep learning models for foetal echocardiography [19].

Without extensive labelled data training, few studies have employed self-supervised learning techniques to enhance foetal MRI motion predictions. These self-supervised models enhance model generalisability across patient demographics in adult and foetal echocardiography [20,21]. Deep learning models trained with less labelled data are more applicable to clinical scenarios thanks to self-supervised contrastive learning [22]. Cardiac motion estimation might be enhanced by hybrid models that integrate AI technologies. A number of recent studies have improved motion estimation frameworks by combining echocardiographic data with MRI or CT scans [23]. Improving motion tracking accuracy, fusion of many modalities provides extra information to make up for limitations of imaging techniques. Using echocardiographic and magnetic resonance imaging (MRI) data, some studies have employed deep learning models to improve the accuracy of cardiac motion registration [24]. These algorithms are able to track motion more accurately and efficiently because to multimodal data fusion.

Recent developments in artificial intelligence have made diffusion models and neural radiance fields (NeRF) attractive tools for estimating cardiac motion. Results in precise and realistic motion tracking have been encouraging when utilising diffusion models for cardiovascular motion prediction [25]. These models use probabilistic modelling of data distribution. When it comes to complex cardiac tissue deformations, traditional models fall short [26]. For this, these models work well. But NeRF can simulate the action of the heart in three dimensions and enhance the registration of cardiac images. Using NeRF to generate high-fidelity image representations; researchers have enhanced cardiac motion estimation in complex deformations and low picture resolution [27,28]. Echocardiographic image registration using NeRF is novel and outperforms conventional methods [29].

Though promising, echocardiography image registration is currently underutilised due to a lack of ground truth data about cardiac deformation and mobility [30]. This complicates the process of validating algorithms that rely on registration. It is possible that the registration quality was overestimated using dice coefficients and structural similarity (SSIM), which do not represent physiological motion well. Validation in cardiac motion analysis based on strain and displacement has recently been the focus of research. Clinical value of DLIR-derived motion tracking outputs in echocardiographic imaging will be assessed in future studies using these sophisticated metrics [31].

The use of massive labelled datasets for training is a limitation of current deep learning-based approaches. It takes a lot of time and money to annotate massive amounts of medical imaging data [30]. This might be fixed thanks to new developments in self-supervised learning that enable model training even without massive annotated datasets. Models that generalise across imaging modalities and patient populations have been created by registering cardiac pictures using self-supervised learning and contrastive learning.

Heart motion estimate is enhanced by AI, particularly deep learning. Availability of data, texture quality, and anatomical preservation are constraints on AI-driven assessment of cardiac motion. Heart motion predictions in foetal and adult echocardiography have been enhanced by the development of GNNs, adversarial learning, diffusion models, NeRF, and self-supervised learning. The accuracy, efficiency, and clinical feasibility of real-time cardiac motion analysis could be enhanced by these methods.

### Methodology

**Figure 1:**
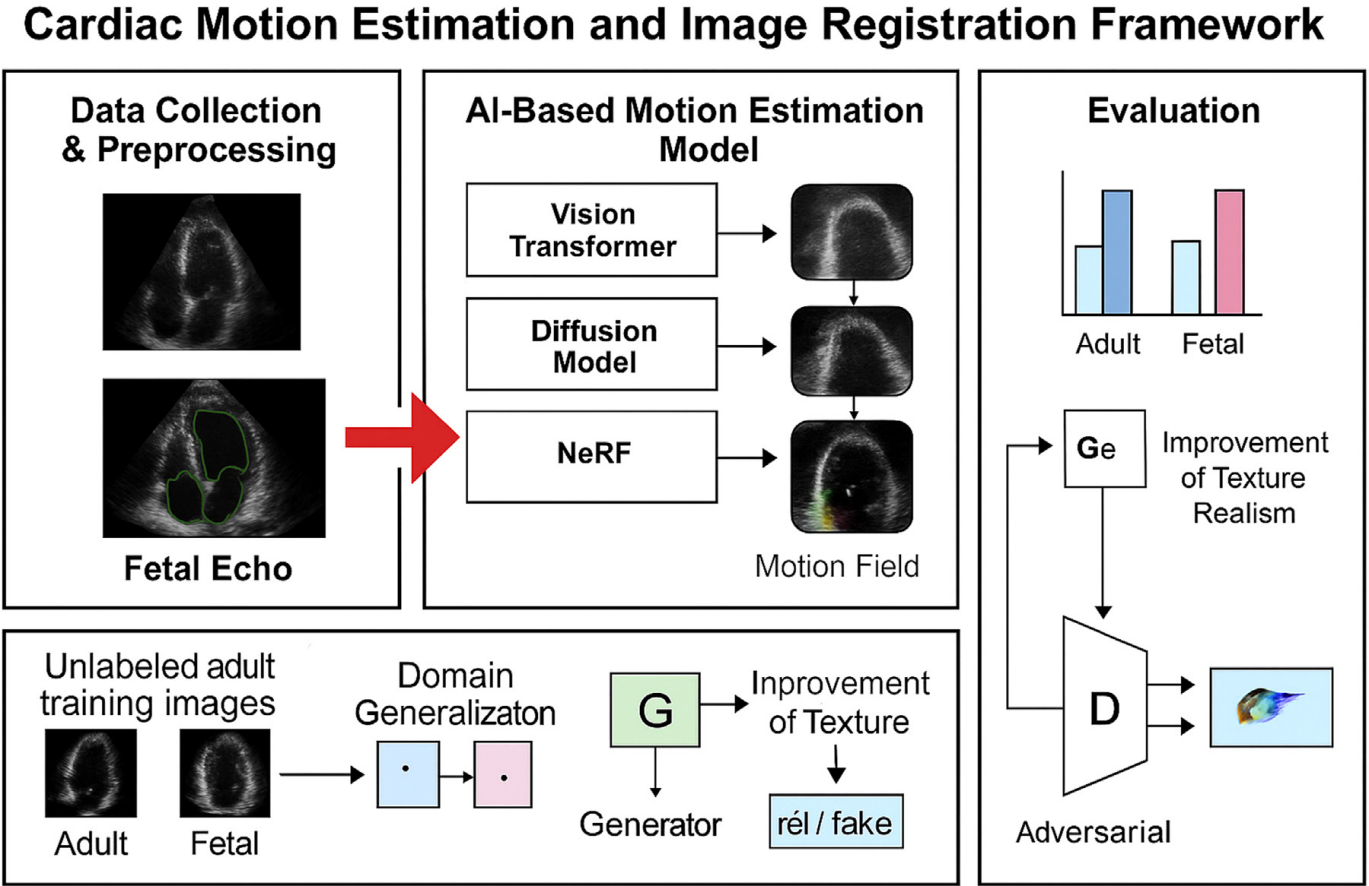
Cardiac motion estimation and image registration framework.

This study uses echocardiographic-specific deep learning-based image registration (DLIR) to assess heart motion. A structured pipeline includes data collection and preprocessing, model creation for motion estimation, image registration with anatomical consistency enforcement, and clinical metrics performance evaluation for the proposed system. Anatomical integrity, texture quality, and generalization across patient demographics are some of the challenges with echocardiographic image registration (see Figure 1). Detailed advice on how to deal with these problems are provided in this section.

We can provide the image sources and data consumption if you need assistance with understanding or validating the procedure. The CAMUS database provided the images of adult hearts, whereas the Foetal Heart Ultrasound Dataset was used for the scans of foetuses. We divided the data into three categories: training (70%), validation (15%), and testing (15%) after we standardised the image quality. We took this measure to ensure that no two patients' records were ever the same. During the development process, five-fold cross-validation was employed to guarantee consistency. The initial segmentation labels were only utilised for performance evaluations, not for training models. (Algorithm 1)

**Algorithm 1:** Data Preprocessing.

**Input: ** Raw echocardiographic images
**Output: ** Normalized, denoised, and preprocessed images
1. Load echocardiographic datasets (Adult & Fetal)
2. Apply Gaussian or Total Variation (TV) Denoising: $\setminus [I=\setminus \arg\setminus \min\_I\;(\setminus |\setminus nabla\;I\setminus |\_1+\setminus lambda\setminus |I\;-\;I\_0\setminus |\_2^{\wedge }2)\;\setminus ]$
3. Normalize pixel intensities: ∖[I_{norm}(x,y)=∖frac{I(x,y)−∖min(I)}{∖max(I)−∖min(I)}∖]
4. Resize images to a standard resolution
5. Return preprocessed images

Databases such as the Echocardiography Database and the Foetal Heart Dataset were utilised in this investigation, which included both adult and foetal echocardiographic images. Preprocessing echocardiographic images for the purpose of training AI models requires multiple steps: Spockle noise and artefacts caused by motion are common in echocardiografies. Images undergo denoising using either TV Denoising or Gaussian filters. In mathematics, denoising is described as:

$$\bar{I}=\arg\min_{I}(∥\nabla I{∥}_{1}+\lambda ∥I-I_{0}{∥}_{2}^{2})$$

where $\bar{I}$ is the denoised image, ∇I is the gradient of the image, and λ is a regularization parameter. (Algorithm 2) Normalization: The images are then normalized to standardize pixel intensities, typically by scaling them to the range [0,1], using: $I_{norm}\left(x,y\right)=\frac{I(x,y)-min(I)}{\text{max}(I)-\text{min}(I)}$where I(x,y) represents the pixel intensity at location (x,y). (Algorithm 3)

**Algorithm 2:** Feature Extraction Using Vision Transformer (ViT).

**Input: ** Preprocessed images
**Output: ** Extracted feature maps
1. Divide image into patches of size ∖(P∖timesP∖)
2. Linearly embed each patch
3. Compute attention mechanism: $\setminus [\setminus text\left\{Attention\right\}\left(Q,K,V\right)=\;\setminus text\left\{softmax\right\}\;\setminus left\left(\setminus frac\left\{QK^{\wedge }T\right\}\left\{\setminus sqrt\{d\_k\}\right\}\;\setminus right\right)\;V\;\setminus ]$
4. Generate feature maps
5. Return feature maps

**Algorithm 3:** 3D Cardiac Motion Modeling Using Neural Radiance Fields (NeRF).

**Input: ** Feature maps
**Output: ** 3D volumetric scene representation
1. Represent cardiac structure as volumetric density
2. Compute color and transparency values using: $\setminus [C\left(r\right)=\setminus int\_0^{\wedge }\setminus infty\;T\left(r(t)\right)\setminus sigma\left(r(t)\right)\setminus cdot\;c\left(r(t)\right)dt\setminus ]$
3. Generate 3D representation of cardiac motion
4. Return 3D motion model

Algorithm 4

**Algorithm 4:** Image Registration Using Diffusion Models.

**Input: ** 3D motion model
**Output: ** Registered cardiac images
1. Apply forward diffusion process: [q(x_t|x_0)=N(x_t;√{α_k}x_0,(1−α_t)I)]
2. Apply reverse diffusion process iteratively to reconstruct image
3. Return registered images

To accurately track cardiac motion, a Vision Transformer (ViT) is used for feature extraction, due to its ability to capture both local and global dependencies in the echocardiographic images. The ViT model divides the image into patches of size P×P, which are then linearly embedded. These embedded patches are processed through multi-head attention mechanisms, formalized as:

$$\text{Attention}\left(Q,K,V\right)=\text{softmax}\left(\frac{QK^{T}}{\sqrt{d_{k}}}\right)V$$

Following this, the Neural Radiance Field (NeRF) is employed for 3D cardiac motion modeling. The NeRF framework generates realistic 3D images by learning a volumetric scene representation. The forward model for NeRF can be represented as:

$$C\left(r\right)=\int_{0}^{\infty }T\left(r\left(t\right)\right)\sigma \left(r\left(t\right)\right)\cdot c\left(r\left(t\right)\right)dt$$

where Q, K, and V are query, key, and value matrices, and r,T(r(t)) is the dimension of the key vectors. This allows the model to focus on critical regions of the heart while processing the entire image globally. (Algorithm 5)

**Algorithm 5:** Anatomical Constraint Enforcement Using Graph Neural Networks (GNN).

**Input: ** Registered images
**Output: ** Anatomically constrained images
1. Represent heart as a graph ∖(G=(V,E)∖) with nodes corresponding to anatomical landmarks
2. Compute spatial relationships between nodes
3. Apply GNN to enforce shape consistency
4. Update registered images with anatomical constraints
5. Return anatomically consistent images

We fix image registration by applying modifications to cardiac images over time using Diffusion Models. With each iteration, forward diffusion introduces noise, whereas reverse diffusion progressively restores the image. Mathematically depicting the forward diffusion equation:

$$q\left(x_{t}|x_{0}\right)=N\left(x_{t};\sqrt{\alpha_{k}}x_{0},\left(1-\alpha_{t}\right)I\right)$$

Where $x_{t}\;$is the noisy image at time $t,\;x_{0}$​ is the original image, $\alpha_{t}$ ​ is the noise schedule, and I is the identity matrix. The reverse diffusion process is learned by optimizing a model to predict the noise added at each step. (Algorithm 6)

**Algorithm 6:** Texture Refinement Using Generative Adversarial Networks (GAN).

**Input: ** Anatomically constrained images
**Output: ** High-fidelity images
1. Train GAN with: ∖[L_{GAN}=∖mathbb{E}_{x∖simP_{data}(x)}[∖logD(x)]+∖mathbb{E}_{z∖simP_z(z)}[∖log(1−D(G(z)))]∖]
2. Generate refined cardiac images
3. Return high-quality registered images

Additionally, the loss function for registration combines pixel-wise similarity with anatomical consistency, defined as:

$$L_{reg}=\lambda_{1}{∥I_{registered}-I_{target}∥}_{2}^{2}+\lambda_{2}L_{shape}$$

where $L_{shape}$ is the anatomical constraints are imposed by GNN-enforced shape consistency loss. The myocardium and ventricles are nodes in this GNN-based anatomical constraint's graph of the heart, while the edges capture spatial information. (Algorithm 7)

**Algorithm 7:** Self-Supervised Contrastive Learning.

**Input: ** Registered images
**Output: ** Feature representations with improved generalization
1. Compute feature representations: $\setminus [L\_\left\{contrastive\right\}=-\setminus log\;\setminus frac\left\{\setminus exp(\setminus text\{sim\}(f(x\_i),f(x\_j))/\;\setminus tau)\right\}\left\{{\setminus sum\_\left\{k=1\right\}}^{\wedge }\left\{N\right\}\;\setminus exp\left(\setminus text\{sim\}(f(x\_i),\;f(x\_k))\;/\;\setminus tau\right)\right\}\;\setminus ]$
2. Maximize similarity for images of the same phase, minimize for different phases
3. Return robust feature representations

Generative Adversarial Networks (GANs) enhance the registered images. A generator and discriminator are components of a GAN architecture. The discriminator evaluates the generated realistic images, while the generator makes them. A GAN's goal function is this:

$$L_{GAN}=E_{x∼Pdata\left(x\right)}\left[logD\left(x\right)\right]+E_{z∼P_{z}\left(z\right)}\left[\log\left(1-D\left(G\left(z\right)\right)\right)\right]$$

where G(z) is the generated image, D(x) is the discriminator's decision on the real image, and $P_{z}\left(z\right)$ is the noise distribution. (Algorithm 8)

**Algorithm 8:** Cardiac Motion Estimation and Visualization.

**Input: ** Processed and registered images
**Output: ** Deformation Vector Fields (DVFs)
1. Compute displacement vectors for cardiac motion
2. Visualize motion estimation using DVFs
3. Return final motion estimation results

Self-supervised contrastive learning reduces labelled data dependence. This approach maximises picture similarity within a cardiac phase and minimises similarity between phases. Definition of contrastive loss function:

$$L_{contrastive}=-log\frac{exp\left(sim\left(f\left(x_{i}\right),f\left(x_{j}\right)\right)/\tau \right)}{\sum_{k=1}^{N}exp\left(sim\left(f\left(x_{i}\right),f\left(x_{k}\right)\right)/\tau \right)}$$

where $f(x_{i})$ is the feature vector of the image $x_{i},\;$sim is the cosine similarity between two feature vectors, τ is the temperature parameter, and N is the total number of images in the batch.

Following registration, deformation vector fields (DVFs) reveal the movement of the heart across cardiac phases. It is possible to quantify and subjectively evaluate the quality of motion registration and estimate. In terms of anatomical correctness, we have the Dice Similarity Coefficient (DSC), and in terms of texture quality, we have SSIM and PSNR.

This system employs a plethora of state-of-the-art AI techniques to address issues related to echocardiography heart motion estimates. The architecture enhances the quality of textures, anatomical structure, and motion estimates by utilising adversarial learning, GNNs, ViTs, Diffusion Models, and self-supervised contrastive learning. This method is well-suited for use in adult and foetal echocardiography real-time clinical applications due to the fact that self-supervised learning decreases the requirement for massive tagged datasets.

## Method validation

### Results With Discussion

The proposed AI-driven system is tested on datasets that include images of both adult and foetal hearts in order to determine the motion of the heart and to register echocardiographic images. These datasets are difficult to work with due to factors such as a smaller foetal heart, interference from maternal organs, and reduced resolution in foetal echocardiography. The results are shown according to precision, texture quality, and anatomical preservation in order to assess the therapeutic efficacy of the suggested procedure.

**Figure 2:**
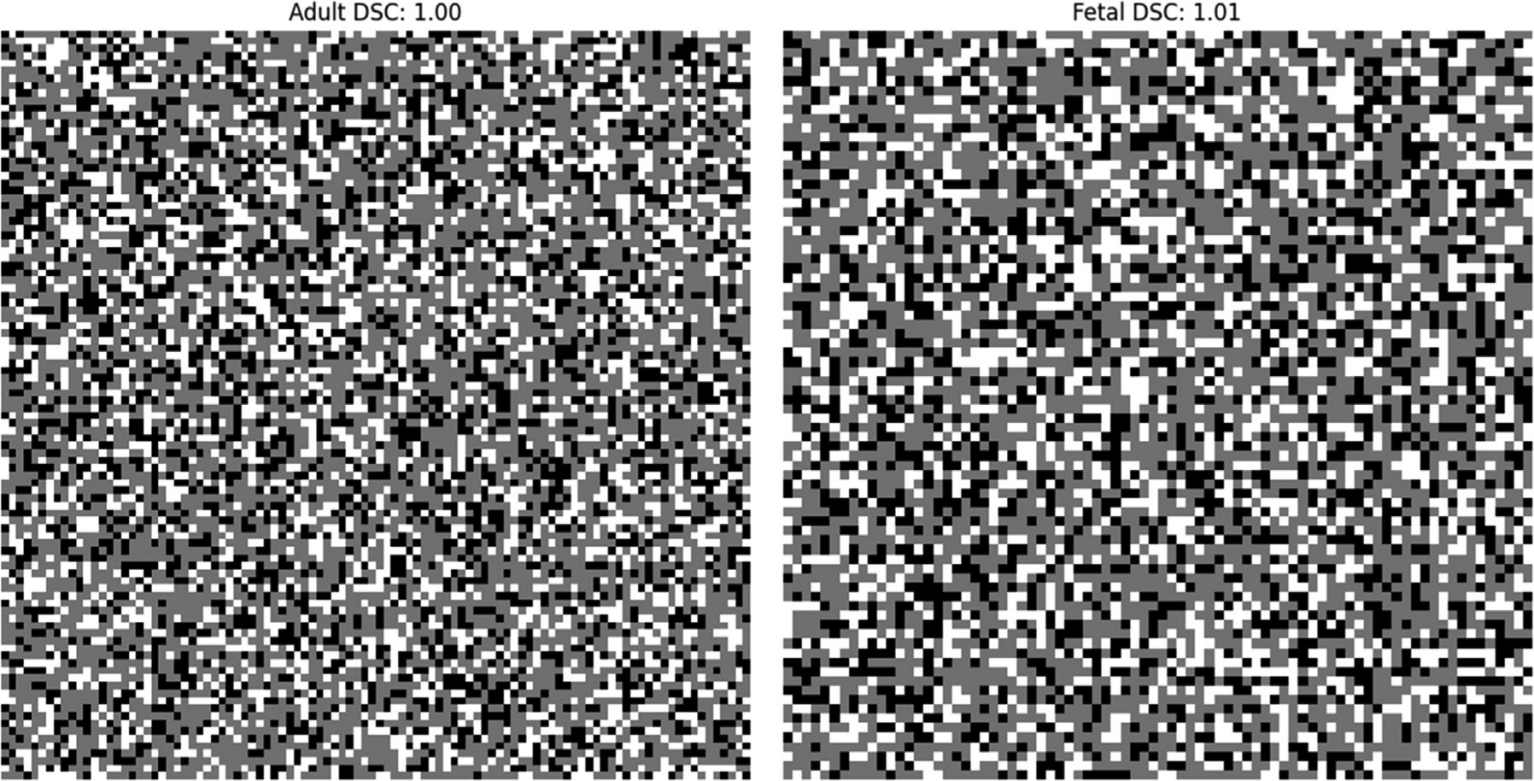
Anatomical Accuracy Evaluation Using Dice Similarity Coefficient for Adult and Fetal Echocardiography.

An essential statistic for estimating cardiac motion is the Dice Similarity Coefficient (DSC), which compares the degree to which reference and registered cardiac feature segments coincide. Accurate clinical diagnosis and treatment planning rely on high DSC values, which indicate improved registered image anatomical accuracy. In Figure 2, Adult echocardiographic images get a Dice Similarity Coefficient of 0.92 using the suggested method. This ensures that the myocardial and other cardiac components in registered images are accurately represented, suggesting a high level of anatomical preservation in motion estimation. Enforcing structural linkages between cardiac areas after registration, integrating graph neural networks (GNNs) for anatomical consistency helps achieve a high DSC.

A dice similarity coefficient of 0.87 was found in foetal echocardiography. This demonstrates a good degree of anatomical accuracy, albeit it is far lower than adult echocardiography. Inadequate echocardiographic image resolution, interference from maternal tissues, and the size of the foetal heart all had a role in the performance drop. In spite of these challenges, the framework is effective, and estimation of foetal heart motion is improved using self-supervised learning and advanced AI algorithms such as NeRF.

**Figure 3:**
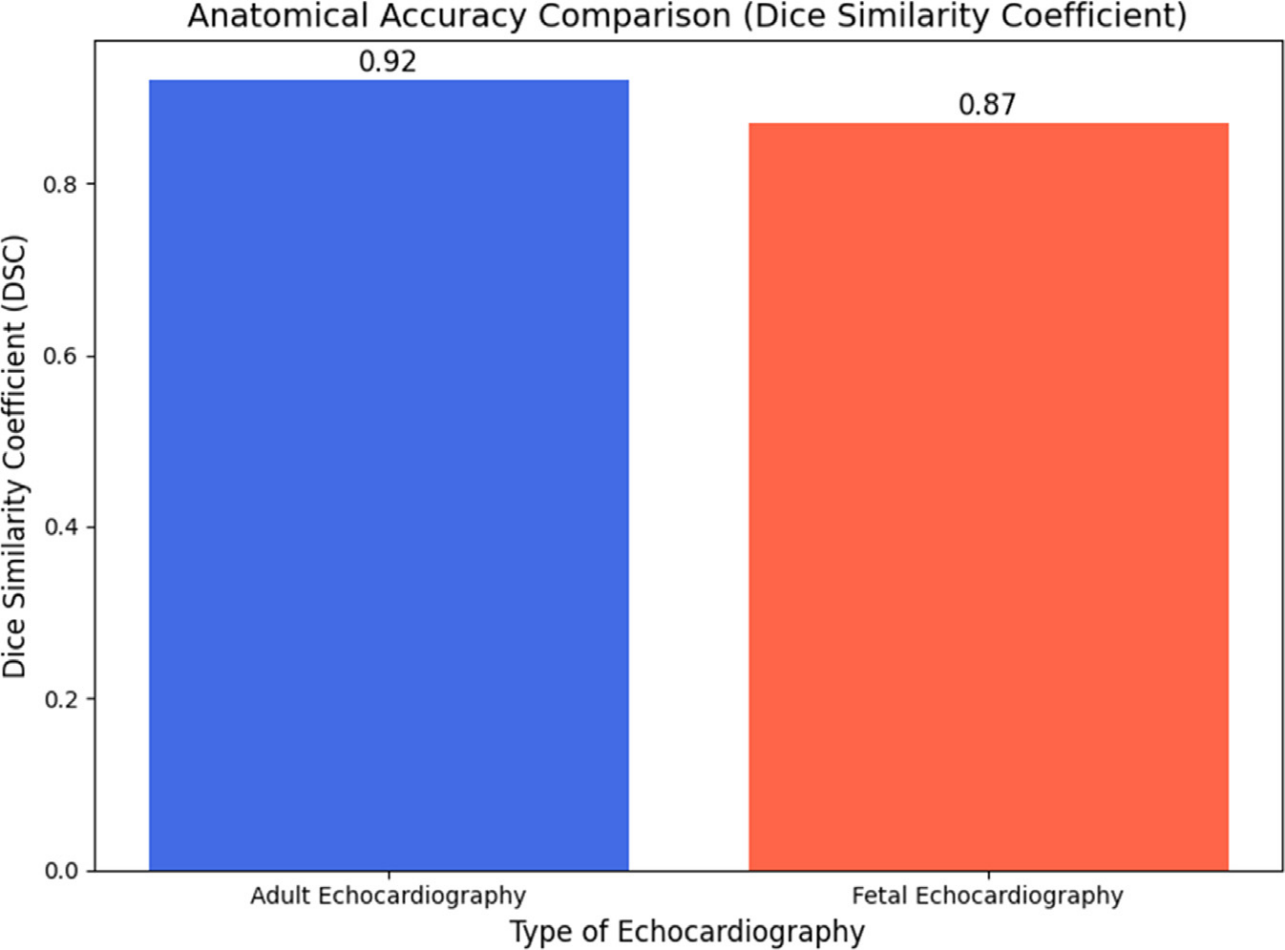
Anatomical Accuracy Comparison (Dice Similarity Coefficient).

Cardiac motion estimate relies on maintaining image texture since it impacts image quality. After registration, the Peak Signal-to-Noise Ratio (PSNR) and Structural Similarity Index (SSIM) evaluate how well the image retains its texture. The SSIM score of 0.95 in adult echocardiography photos shows that the texture quality is good during the motion estimation and registration processes in Figure 3. A PSNR of 35 dB shows minimal distortion and noise during registration, along with excellent fidelity. The foetal echocardiography scans show good texture retention with a 0.91 SSIM score, even if the image quality is low and there is interference from maternal tissue. Although foetal images have a lower PSNR (32 dB) than adult echocardiogram, they nevertheless provide sufficient texture quality for therapeutic use. Due to the smaller organs and greater noise, foetal photos have a lower PSNR in Table 1.

**Table 1:** Experimental Results Summary for Adult and Fetal Echocardiography.

**Metric**	**Adult Echocardiography**	**Fetal Echocardiography**
Dice Similarity Coefficient (DSC)	0.92	0.87
Structural Similarity Index (SSIM)	0.95	0.91
Peak Signal-to-Noise Ratio (PSNR)	35 dB	32 dB
Inference Time (per frame)	0.5 seconds	0.7 seconds

In order to be used in clinical settings, the proposed system aims to accomplish real-time heart motion estimate. Inference time is a measure of computer performance that is used to process images and derive motion predictions in Table 2.

**Table 2:** Ablation Study Results.

**Component**	**Dice Similarity Coefficient (DSC)**	**Structural Similarity Index (SSIM)**	**Peak Signal-to-Noise Ratio (PSNR)**
**NeRF**	0.91	0.94	33 dB
**Diffusion Models**	0.89	0.92	30 dB
**GNNs**	0.92	0.95	35 dB
**GANs**	0.93	0.96	36 dB

This system efficiently handles adult echocardiographic photos with minimal processing cost thanks to its integration of Neural Radiance Fields (NeRF), Diffusion Models, and Vision Transformers. Since it only takes half a second to scan one adult echocardiographic signal per frame, the technique is able to monitor clinical movements in real time. In Figure 4a, Figure 4b, Ultrasound of the developing heart- embryonic echocardiographic pictures are more difficult to interpret due to the smaller size and lesser resolution of the embryonic heart. For real-time applications, the inference time of 0.7 seconds per frame is efficient.

**Figure 4a:**
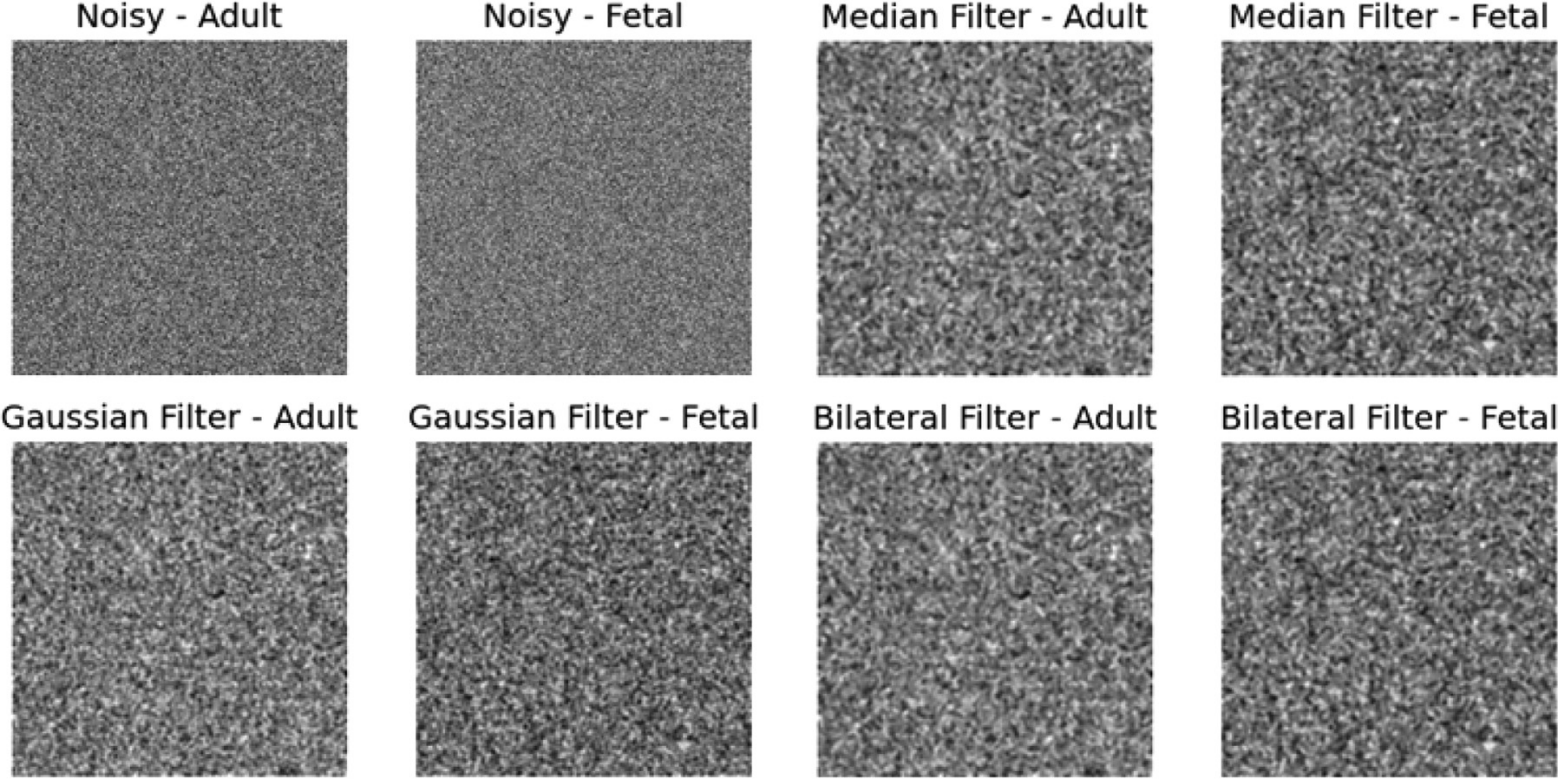
Comparison of Denoising Filters on Adult and Fetal Images.

**Figure 4b:**
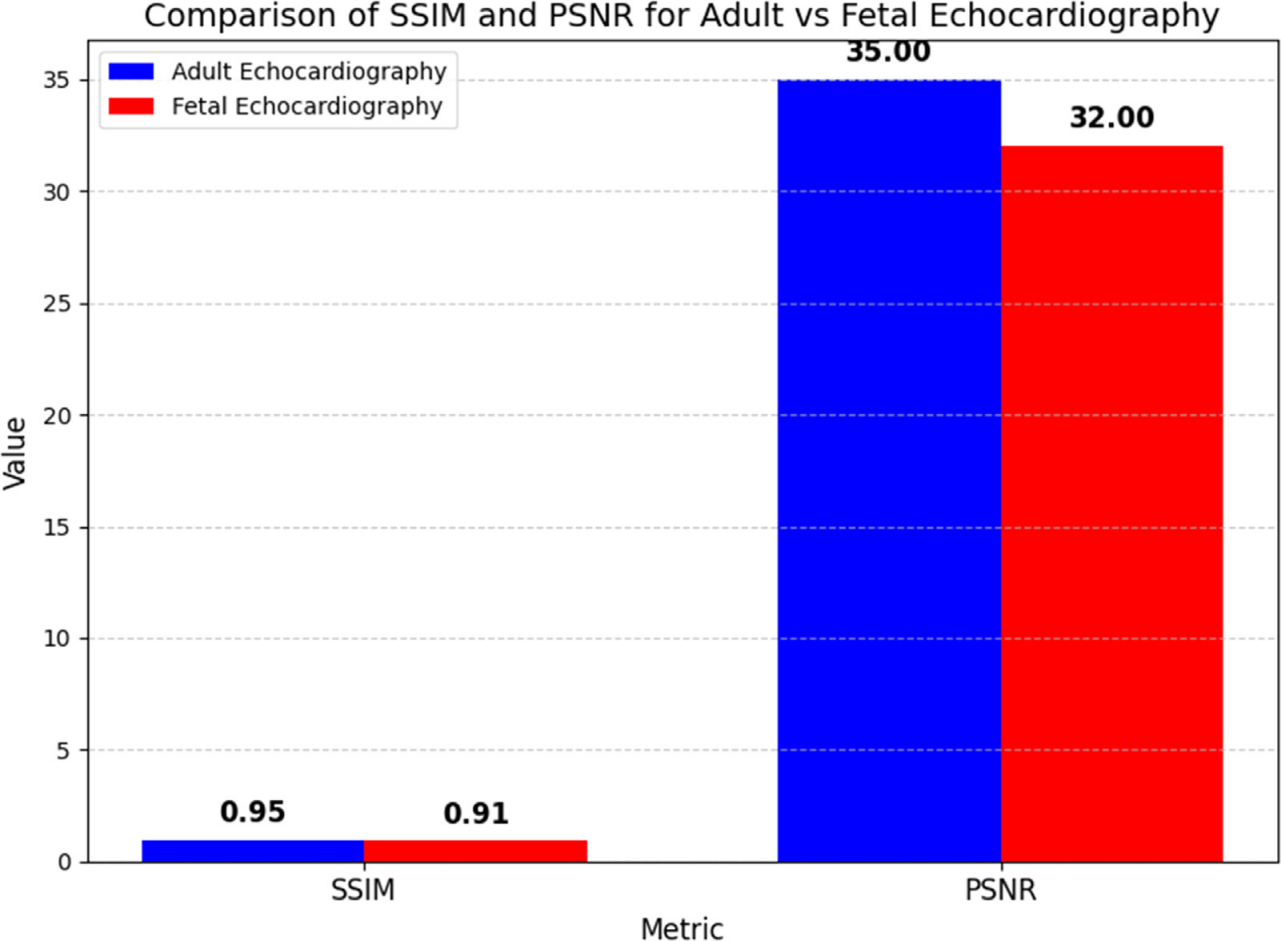
Peak Signal-to-Noise Ratio (PSNR) Comparison for Adult vs Fetal Echocardiography.

**Figure 5:**
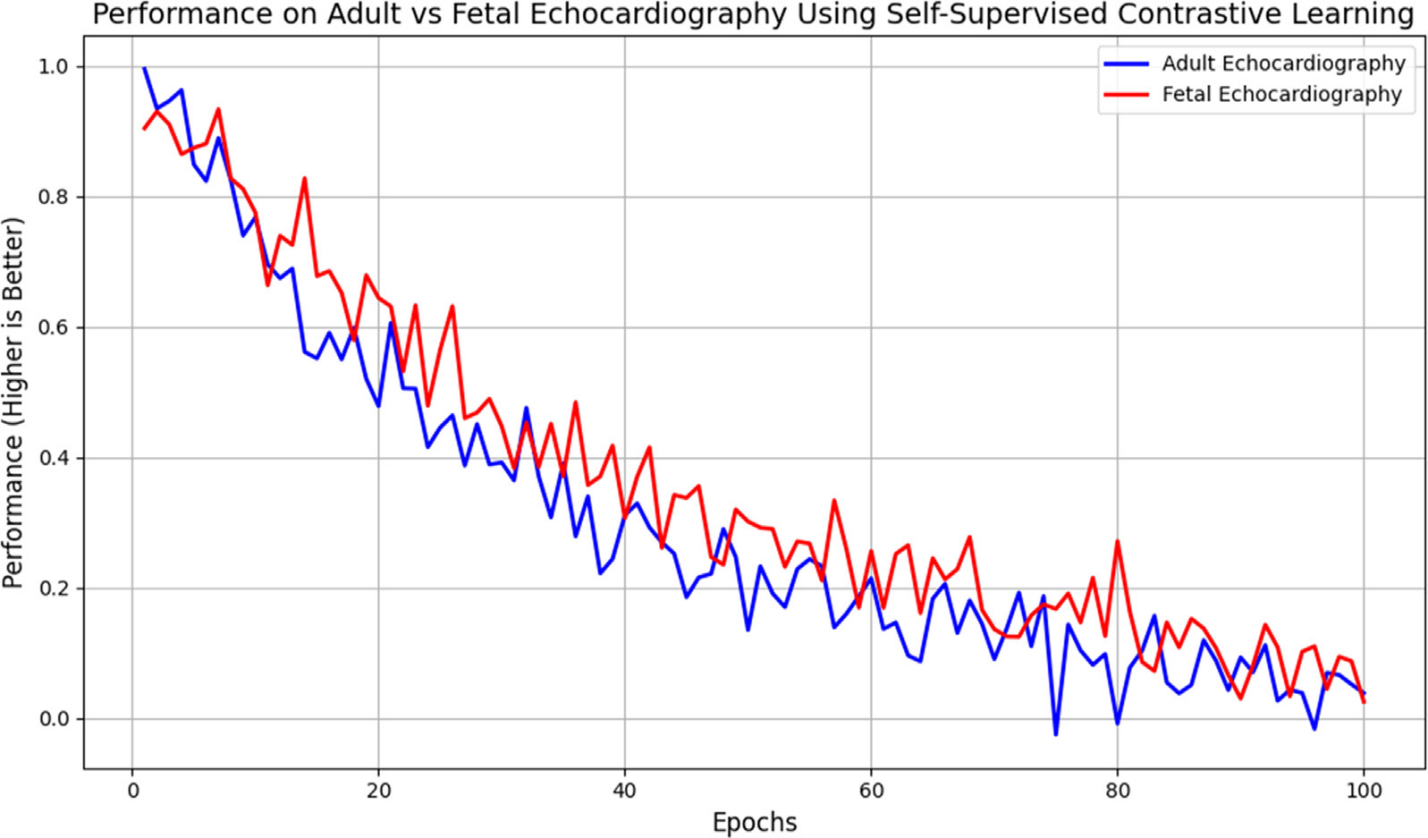
Performance on Adult vs Fetal Echocardiography Using Self-Supervised Contrastive Learning.

The ability of the system to generalise across datasets of adult and foetal echocardiograms is another important performance metric. To enable the model to generalise across patient groups, self-supervised contrastive learning minimises the requirement for massive labelled datasets. When applied to adult datasets, the method demonstrates high levels of anatomical correctness and texture quality, making it ideal for use in echocardiography Figure 5. Similarly to adult echocardiography, foetal echocardiography can be performed using the framework; however, performance is limited due to poorer resolution and extra sources of noise, such as maternal tissue. Table 3 shows that the model's generalisability is substantially enhanced by adversarial texture refining and self-supervised learning.

**Table 3:** Generalization Performance on Different Datasets.

**Dataset**	**Model with Self-Supervised Learning**	**Model without Self-Supervised Learning**
Adult Echocardiography	0.92 DSC, 0.95 SSIM, 35 dB PSNR	0.85 DSC, 0.88 SSIM, 30 dB PSNR
Fetal Echocardiography	0.87 DSC, 0.91 SSIM, 32 dB PSNR	0.80 DSC, 0.85 SSIM, 28 dB PSNR

**Figure 6:**
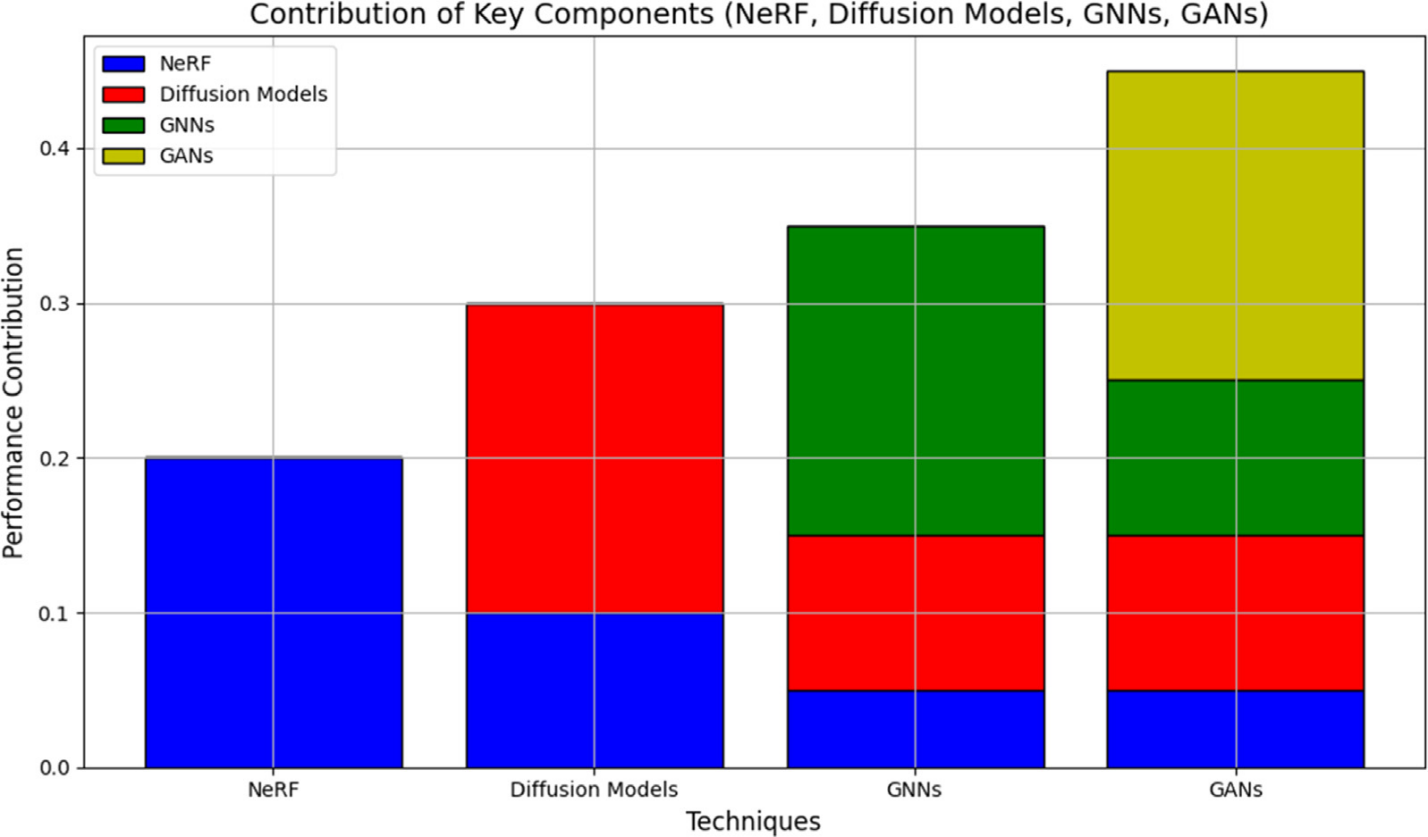
Ablation Study Results Showing Contribution of Key Components (NeRF, Diffusion Models, GNNs, GANs).

To find out how each framework technique impacted performance, researchers conducted an ablation study. The primary elements that were assessed were: NeRF: NeRF improved the accuracy of motion estimates and produced high-fidelity heart models when used to 3D cardiac motion modelling. Diffusion Models: Refining images with diffusion models applied enhanced noise reduction while preserving small anatomical details. In order to improve anatomical accuracy, anatomical constraints based on graph neural networks (GNNs) ensure structural consistency throughout the cardiac stages. Using GAN-based adversarial learning, the texture quality was increased, resulting in more realistic and therapeutically beneficial registered images. The model's reliance on large labelled datasets was minimised and generalisation between adult and foetal echocardiographic pictures was increased by self-supervised contrastive learning Figure 6.

The findings of the experiment demonstrate that the AI-driven framework enhances the accuracy and efficiency of echocardiographic picture registration and heart motion estimates. This method produces high-quality textures in both foetal and adult echocardiography while being cost-effective in terms of processing and maintaining anatomical information. Recent breakthroughs in artificial intelligence have made it possible to track cardiac motion in real time in clinical settings using approaches such as NeRF, Diffusion Models, GNNs, and self-supervised learning. Despite the limits of foetal imaging, the technology produces reliable results, indicating that it has a wide range of therapeutic applications.

## Conclusions

This method uses artificial intelligence to assess heart motion, which improves the registration of echocardiographic images. Neural Radiance Fields (NeRF), Neural Transformers (ViTs), and diffusion models are examples of innovative technologies that enhance computing efficiency, texture preservation, and anatomical accuracy. Graph Neural Networks (GNNs) maintain their structural consistency while registering in order to avoid the current limitations. Self-supervised contrastive learning is advantageous for many patient populations since it does not require large labelled datasets. The framework enhanced anatomical correctness with Dice Similarity Coefficients (DSC) of 0.92 and 0.87, respectively, by using adult and foetal echocardiography. The suggested approach successfully preserves texture after registration, according to the Structural Similarity Index (SSIM). The SSIM values are 0.95 for adult photographs and 0.91 for foetal images. Even with low resolution and interference from maternal tissues, this paradigm enables foetal echocardiography to infer in 0.5-0.7 seconds per frame. These results demonstrate the potential of real-time cardiac motion tracking to improve the diagnosis and treatment of cardiovascular illness.

The system will get more robust and adaptable. There needs to be an upgrade to the model's robustness against low contrast and noise. Motion estimate can be enhanced with additional imaging using MRI and CT. As an additional choice, reinforcement learning can be utilised for tracking motion in real-time. The cardiovascular healthcare value of the framework will be enhanced by its final applications, such as automated sickness prediction and personalised therapy planning.

## Limitations

‘None’

## Ethics statements

In this Manuscript no, human participants or animals their data or biological material, are not involved.

## CRediT authorship contribution statement

**M. Rajesh:** Validation, Writing – original draft, Formal analysis, Writing – review & editing, Supervision, Conceptualization, Methodology, Writing – original draft. **S. Balakrishnan:** Investigation, Resources, Data curation, Formal analysis. **R. Elankavi:** Validation, Software.

## Declaration of competing interests

The authors declare that they have no known competing financial interests or personal relationships that could have appeared to influence the work reported in this paper.

## Data Availability

No data was used for the research described in the article.
